# “*Sharpening Your Mind, Strengthening Your Body*” Parental Perceptions on the Use of Strength and Conditioning in Children and Youth

**DOI:** 10.3390/children9101557

**Published:** 2022-10-14

**Authors:** Michael Joseph Duncan, Ricardo Manuel Gonçalves Martins, Emma Lisa Jane Eyre

**Affiliations:** Centre for Sport, Exercise and Life Sciences, Coventry University, Coventry CV1 5FB, UK

**Keywords:** resistance exercise, coaching, adolescents, soccer, focus group, qualitative

## Abstract

Although strength and conditioning is beneficial and safe for children to engage in there remain myths and misconceptions form parents regarding its use which prevent its widespread take up. This study explored parents’ attitudes and beliefs about strength and conditioning in their children. Thirty-one parents (21 dads, 10 mums) took part in one of four focus groups exploring the topic. Thematic analysis was used resulting in themes and sub themes centred on: Beliefs; Determinants; Coach Education; Coach Communication; and Relationship to the Game. There were also smaller aspects of the focus groups which touched upon autonomy as a concept related to implementation of strength and conditioning specifically. Overall, parents of children who play grassroots sport hold generally positive perceptions on use of strength and conditioning with their children, considering it beneficial for both physical and mental development. Key factors relating to successful implementation of strength and conditioning for children focus on having a qualified coach in that particular area (rather than a generic sports coach), effective communication between coach and parents, and coach and children in terms of the benefits of engaging with strength and conditioning.

## 1. Introduction

There is now a considerable body of scientific evidence which demonstrates beneficial effects of strength and conditioning, particularly resistance training in children and youth [1,2]. Such benefits are wide ranging, and include increases in muscular fitness (strength, endurance, power), enhancing fundamental movement skills [3] and motor competence, increase bone mineral density [4], improve cardiometabolic health [5], promote healthy weight [6] and positive mental health [7,8]. Moreover, the benefits accrued from engaging in resistance training during childhood and youth have lifelong benefit as increased bone mineral density in childhood translates to reduced risk of osteoporosis during older adulthood, via impact on peak bone mineral density [4], and improvements in fundamental movement skills create a positive trajectory of health, leading to increased physical activity and lower levels of obesity into adulthood [9].

The evidence related to the benefit of resistance exercise in children and youth that has been published over the last two decades is overwhelmingly positive in nature. In recognition of this, the WHO recommend that children and adolescents participate in activities that are muscle strengthening in nature at least three days per week [10]. Likewise, guidelines for physical activity in the United Kingdom have developed over time with the most recent guidelines emphasising the importance of including muscle strengthening activity at least three days per week [11]. Despite this, literature suggests that children worldwide fail to participate in muscle strengthening activity to the levels recommended [12] and, over the last 50 years, levels of muscular fitness have declined worldwide, including in the UK [13]. This trend is of considerable public health concern as low levels of muscular strength is a recognised risk factor for disability, dysfunction and ill health in older adulthood [1] and recent work by Faigenbaum et al. [14] has suggested contemporary children and youth are equally vulnerable to the consequences of muscle disuse and dysfunction which are avoided via ongoing exposure to specific muscle strengthening exercise.

There is however a key barrier which needs to be addressed to enable children and youth to engage in resistance exercise. According to Faigenbaum et al. [14] the key barrier preventing children engaging in resistance and muscle strengthening exercise is deep seated and unfounded beliefs held by health professionals, teachers, and parents. Specifically, the myth that resistance exercise in unsafe, ineffective, or unnecessary for children and adolescents [14]. Such unfounded beliefs deter adults, including parents, from recommending or facilitating resistance exercise as a potential means to enhance children’s health, well-being and performance [14]. Parental beliefs around strength and conditioning are a key influence for children with recent research, using focus group interviews in a sample of adolescent boys identifying parents as a significant factor in engagement in resistance exercise/muscle strengthening activity in youth [15]. In this aforementioned study, parental attitudes and beliefs and parental support were perceived by boys as key factors in engagement in resistance exercise, including logistical support to assist engagement (e.g., travel to and from the gym) as well as negative attitudes which discouraged engagement (e.g., belief that resistance exercise is unsafe for adolescents, or it will stunt growth) [15]. Adolescents in the study by Cox et al. [15] perceived parents as having no interest in resistance exercise or concerns around regarding potential for injury arising from resistance exercise as a barrier to participation. Consequently, Cox et al. [15] suggested, based on the perceptions of their adolescent participants, that improving parental knowledge relating to the benefit of muscle strengthening activity and dispelling unfounded concerns around resistance exercise might better support future interventions with children and youth. This is a logical suggestion, but is based on perceptions of children themselves, rather than understanding parental views of resistance exercise. Given the considerable volume of literature regarding the importance of parents and parental support in children’s’ physical activity and sport participation particularly [16], it is perhaps surprising that no study to date has examined parental vies/perception/beliefs of resistance exercise in children and youth. As parents represent a critical influence in children’s lives [17] and can both facilitate and impede their children’s engagement in sport and physical activity [18,19], understanding their views/beliefs/perceptions around use of strength and conditioning is a key, needed first step for effective implementation of strength and conditioning with children and adolescents. Examining parental perceptions and beliefs in this respect has also been recommended as a future research need [15]. Without this, evidence-based strategies on how parents can support the implementation of muscle strengthening interventions will remain unknown.

The present study sought to address this noted gap in the literature base by determining and discussing parents attitudes and beliefs about strength and conditioning in their children.

## 2. Materials and Methods

### 2.1. Participants

Following institutional ethics approval and informed consent 31 parents (21 fathers, 10 mothers) of grassroots soccer players took part in this study. To ensure explicit and comprehensive reporting, the consolidated criteria for reporting qualitative research (COREQ) and Standards for Reporting Qualitative Research (SRQR)were used to guide the reporting process [20]. 31 Parents from a potential total of 35 were provided with the study information and invited to participate in the study based on eligibility criteria. All participants were parents of a child aged between 10 and 14 years old who was currently engaged in competitive grassroots football within Birmingham County FA. The definition of grassroots soccer employed in the current study adhered to the FIFA [21] definition as well as aligning with the structure of grassroots soccer in England. To be eligible to participate, participants had be a parent of a child who was registered (and playing) with a grassroots soccer club with at least 1 year playing experience prior to taking part, and including participation in training and organised fixtures against other grassroots teams within the County FA structure in England.

### 2.2. Procedures

The research took a pragmatist approach whereby the research process in the current study recognises that preconceived topics such as views and perceived importance of constructs related to strength and conditioning for children would be reflected in the responses provided by participants. Four purposive semi structured focused groups were arranged and conducted face to face by the final author (Gender: female, credentials: PhD, Occupation: Associate Professor) at the same time their child was undertaking an organised coaching session. Parents were approached to participate as a consequence of their child attending a public participant outreach activity at the university which comprised an educational session for children on fitness assessment for football, followed by a football coaching session. The distribution of focus group numbers and makeup was therefore partly determined by the number of parents willing to participate within each of the given training session slots. Focus groups were homogenous with parents all having children at the same grassroots club (albeit their children played in different teams within the club). Each focus group consisted of between five to nine parents aged between 32–54 years of age. Of 35 potential participants, 31 volunteered to participate (Focus group 1: parents 3 dads: 2 mums, focus group 2: 4 dads: 1 mum, focus group 3: 8 dads: 1 mum, focus group 4: 6 mums: 6 dads). Focus groups lasted between 31 and 51 min (mean = 42.25 min, SD = 7.9) and were conducted in a private space at the University conducting the research. Four parents declined to participate due to having other commitments at the time the focus groups were taking place of for other undisclosed reasons. A semi structured guide (See Table 1) was used to ensure consistency on the topics discussed between each interview but to allow a degree of freedom and adaptability in getting the information from the parents. McNamara’s [22] eight principles of interviewing were followed. Open ended questions which were neutral, clear were developed on the guidance of recommendations for constructing effective questions for focus groups [22]. The open ended questions were aligned to the research questions of understanding what parents perceptions are of strength and conditioning and what factors would need to be addressed in the implementation of strength and conditioning practices for their children. Prior to undertaking the focus group interviews, the open ended questions were piloted with the research team and research questions were refined from the feedback gathered. This is considered an important element in interview preparation [23].

At the onset of each focus group session but prior to the interview itself, relationships between facilitator and parents were established using an introduction, ice breaker activity. Any relationships between the parents participating in the focus group interviews were confirmed at this point with parents stating that they already knew each other. Participants were informed that the facilitator did not have a background or qualification in strength and conditioning but was interested in children’s movement. The goal of conducting the research, to understand what parents’ views and perceptions were regarding strength and conditioning and to gain their views on whether they would want this practice to be implemented, was also made clear to participants.

In regard to research reflexivity, the facilitator has vast experience in focus group interviews and has published multiple articles using this approach. The facilitator engaged in reflexivity prior to conducting the focus groups, during and throughout the analytical/reporting processes and took field notes during the process. Prior to commencing the interviews, the interviewer was aware of their own personal bias from a research perspective with an interest in motor development, their use of resistance training as a form of exercise and their inability to relate as a parent. The interviewer was aware of the myths parents might feel around strength and conditioning particularly related to concerns about the impact it may have on growth and development. Furthermore, the researcher felt that parents may be concerned about the suitability and perceived negative impact of strength and conditioning on their child’s development and risk of injury. The interviewer also felt that many parents might not understand what strength and conditioning, or integrated neuromuscular development may mean and thus developed a powerpoint presentation using a video from the published work of Fort-Vanmeerhaeghe et al. [24] to outline approaches used. The interviewer’s interest in the topic area was to identify what parents may need moving forward if strength and conditioning was to be implemented in their clubs. Throughout the discussions only the facilitator was present.

Throughout the interview, prompts and follow up questions were used to optimise the responses provided [25]. All focus groups were conducted in a quiet environment using a circular seating arrangement. The interviewer facilitated group discussion around the guide and used a mixture of verbal prompts and non-verbal language (e.g., eye contact) to encourage contribution by all individuals and further expansion of responses. Paraphrasing was used and the end of key topic discussions to ensure correct interpretation and clarity and thus removing the need for transcripts to be returned to participants for comment and encouraging member reflections. All focus groups were audio recorded and transcribed verbatim.

### 2.3. Data Management and Analysis

Following anonymisation of the transcripts, employing focus group number and participant number (e.g., FG1, P1), inductive thematic analysis was conducted by one author using the step by step process as described by Braun & Clarke [26,27]. The process resulted in the formation of a thematic map with themes, sub themes, relationships between themes and associated quotes. Throughout the interview, analytical process and reporting of results, field notes, reflexivity and critical friend discussions were held to be transparent about selective and interpretive bias, to debate and re-define themes and develop rigour [28,29]. Data saturation was considered when new data or themes were identified [30].

## 3. Results

**Figure 1:**
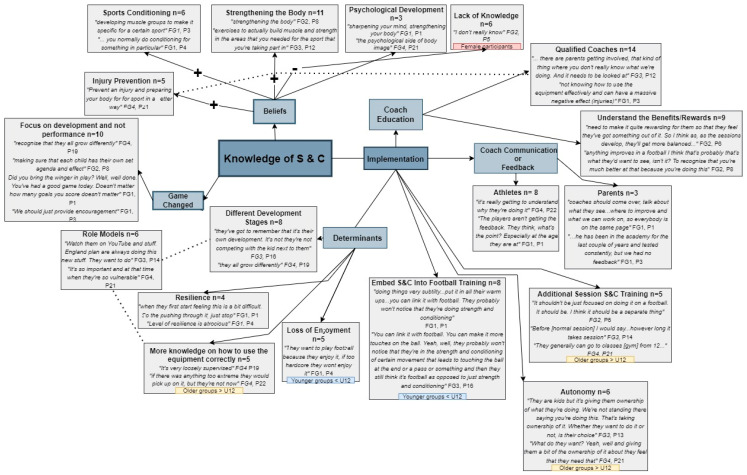
Key themes and sub-themes generated from the focus group discussions reflecting parental knowledge, beliefs and perceptions of strength and conditioning for children.

Results from focus groups centred on parental knowledge and beliefs about strength and conditioning and their perceptions about the implementation of strength and conditioning with their children revealed five main themes with a number of overlapping subthemes. The five themes comprised Beliefs; Determinants; Coach Education; Coach Communication; and Relationship to the Game. There were also smaller aspects of the focus groups which touched upon autonomy as a concept related to implementation of strength and conditioning specifically. These themes and subthemes are visually represented with indicative quotes from focus groups in Figure 1. We also provide an overview with specific quotes in the narrative following Figure 1. Parental perspectives in relation to these themes also appeared to be varied depending on the age stage the parents’ children currently played in with similar themes but different foci where children were playing at Under 12 or below (where the game is focused on mini soccer of 9 vs. 9 formats and smaller) or Under 13 and above (where the game is 11 vs. 11 and the format is similar to adult football). A coding tree for themes is also presented in the Appendix A. Please note, within the coding tree ‘autonomy’ features as a sub theme and ‘role models’ appears as a separate aspect of the focus groups. Comments related to autonomy appeared to be related to the other themes which were identified as a consequence of the focus groups. The concept of role models was identified by parents but stood apart from the other aspects of the discussion and is thus represented as such in the coding tree.

### 3.1. Parent’s Knowledge and Beliefs about Strength and Conditioning

All parents held positive perceptions about strength and conditioning on the whole but recognised positives and negatives for children undertaking it. Dads appeared to have a greater understanding of what strength and conditioning meant and described it as mainly strengthening the body and sports conditioning. The psychological development, both in terms of mental skills for performance and mental well being was also discussed. In relation to what Strength and Conditioning comprised For example:

*“sharpening your mind, strengthening your body” (FG1, P1) and “the psychological side of body image”* (FG4, P21)

Parents perceived Strength and Conditioning to have benefits for injury prevention. For most mothers knowledge of strength and conditioning initially appeared to be lacking, but having seen the prompt video or listened to other views of those in their focus group, felt similarly that it could be a good thing for their child’s development and prevention of injuries. There was a particular discussion that the game of football had changed now and that the focus is much more on development of the whole player/child than football performance as a sole goal. While parents held positive views on the whole there was a discussion on the impact of their children doing strength and conditioning wrongly because their bodies are still growing. They felt that it was important for them to be guided to it in an appropriate way (Figure 1). This is also exemplified in the following quotes from focus groups:

*“And for me, it’s about I see it more of a preventative in terms of getting them. The more core strength and that they’ve got and the better fitness they’ve got. The less likely they are to injure themselves because I think they’re less likely to over stretch themselves when they’re playing, so they’ve got that core strength. So yeah, I’m very keen that yeah.”* (FG4, P19)

*“Yeah, but I think it’s important that clubs do it to guide them.”* (FG4, P19)

*“I think there’s pros and cons of it. Because the pros that we’ve already spoke about a massive advocate of it, but there’s also now a lot of equipment, that’s now put into a lot of sports. And if you don’t necessarily know how to use that equipment, effectively, I think it can have a massive negative effect …, and if you put the wrong one [child] with the wrong level, that can then have an effect on could have an effect on the muscle growth, or kind of the stature of someone’s posture or so I think it can have a negative effect if somebody’s not trained …”* (FG1, P3)

### 3.2. Parent’s Perceptions about the Implementation of Strength and Conditioning with Their Child

Parents held differing views about how strength and conditioning should be implemented based on the ages of their children and in relation to their engagement in organised sport. For those in the U12′S group, there was a feeling that it should be embedded and integrated within their regular football training such as the warm-up so it is hidden and the children are not explicitly aware that they are doing this type of training. This was associated with the concerns around loss of enjoyment, that the sessions might become too ‘hardcore’ with one parent stating: *“They want to play football because they enjoy it, it too hardcore they won’t enjoy it”* (FG1, P4) which was agreed with by a number of parents and with concerns that employing strength and conditioning without integration into football would therefore result in lower levels of enjoyment. For example:

*“The only way you’d be able to do it is by putting a new condition practice in the middle and just put little conditions into a game or different equipment into it. So, if you went through gates, you’d score an extra point, if you hopped over something if you did. So little conditions. Yeah. But little conditions, gain kind of specific drills. And again, but then you’re doing things very subtly. And then it comes back to the psychology then they probably won’t notice that they’re doing strength and conditioning rather than switching them off.’…’Yeah, I’ll try and put it in all their warm ups. So it’s a little bit more discreet. So you can make it a little bit competitive, but also in the warm up. So they’re still getting constant 10, 15 min every session. And then they’ve still got them the technique.”* (FG1, P1)

Parents also acknowledge that strength and conditioning might implicitly link to football performance of their children as well, as demonstrated by this quote:

*“You can link it with football. You can make it more touches on the ball. Yeah, well, they probably won’t notice that they’re in the strength and conditioning of certain movement that leads to touching the ball at the end or a pass or something and then they still think it’s football as opposed to just strength and conditioning.”* (FG3, P16)

Where parents had children playing in older age groups (U13s–U16s), parents felt that additional sessions should be held instead of integrating them within the football session. Parents reported here how some of the children were already engaging in strength training outside of the training sessions either at home or the gym facilities but that they need more knowledge on how to use the equipment correctly (See Figure 1). The context to this is exemplified by the following quotes:

*“It shouldn’t be just focused on doing it on a football. It should be. I think it should be a separate thing”* (FG2, P6)

*“It is changing the idea of strength training isn’t. It’s not. It’s not just not weighted slots, and it lunges and it’s all. It’s body weight. It’s about the right time, and yeah doing body resistance and teaching them how to do it safely. I think is really important and my older one, you know. He’s he’s been going to the gym and that’s always been. My concern is that he’s pushing himself. Yeah, he’s he’s now he’s a rugby player, so there’s even more pressure on that.”* (FG4, P19)

There was also an acknowledgement that social connectedness to the team may facilitate social norms which need consideration when a group are starting to engage in strength and conditioning. The segment below form one of the focus groups illustrates this point:

*“…in* [MUTED CLUB NAME] *they’re all started to go to the gym ‘cause allowed to go through teams so they’re all starting to use the little gym...”* (FG4, P22)

*“Why they’re doing what they’re doing, what they’re trying to prevent with injuries down the line and recognising their limitations as well because what one person can do doesn’t mean the another one can.”* (FG4, P21)

*“Yeah, and also to recognise that they all grow it differently…And there you know, and recognise when they should stop, say what. What’s a good hurting bit? What’s baking ache good vs. a bad ache”* (FG4, P19)

*“The ideal is that they want them to go to that so that it can learn how to. Do it properly and. Then progress up to the gym and that’s how they started it, but I think.”* (FG4, P19)

Across all parents and age groups, it was believed that having a qualified coach who communicates and provides feedback to both the parents and the athletes as well as ensuring athletes understand the benefits and rewards ‘feel they got something out of the session’.

For example, in regard to communication from the coach to parents:

*“Coaches should come over, talk about what they see … where to improve and what we can work on, so everybody is on the same page”* (FG1, P1)

*“…he has been in the academy for the last couple of years and tested constantly, but we had no feedback”* (FG1, P3)

Furthermore, in regard to communication from the coach to the children/players themselves:

*“If the players aren’t getting the feedback. They think, what’s the point? Especially at the age they are at.”* (FG1, P1)

*“It’s really getting [the child] to understand why they’re doing it”* (FG4, P22)

Parents also shared how children are watching their role models, such as professional footballers, on YouTube and social media platforms and how these are key influencers and could be used to promote knowledge and behaviour. For example:

*“Watch them* [The England national team] *on YouTube and stuff. England players are always doing this new stuff. They want to do.”* (FG3, P14)

The main concerns expressed by parents around implementation of strength and conditioning were focused on who was doing it, it was felt that this needed to be done by someone suitably qualified and where the different development stages could be considered, otherwise parents feared it could have a negative effect. This is exemplified by the quote below:

*“I think it’s perhaps important to understand that children certainly at this age, develop at different rates as well. Yeah. So, the level of like the training that would need to be sort of, not necessarily tailor-made to an individual child level, but broad enough to meet the needs of groups of children as opposed to vote, you know, just a blanket overall process.”* (FG3, P11)

And:

*“My first thoughts. There is like being involved with them is like you really need someone that knows what they’re doing before we embark on this sort of thing.”* (FG4, P22) *+ confirmed by* (FG4, P21-P18-19)

In this regard, parents knowing the coach of their child’s team also appeared to be an important consideration, for example:

*“We all know how* [S & C COACH NAME] *works. And we all know, his professionalism, we all know our previous jobs. In terms of being run through the club that he’s involved in, I wouldn’t have an issue at all. I’d have an issue Sunday morning football, and it was, right, this is what you’re doing? Yeah. Like you say, there are parents getting involved, that kind of thing where you don’t really know what we’re doing. And it needs to be looked at”.* (FG3, P12)

The above quote was then followed by:

*“That comes down to quality again, doesn’t mean you got somebody who knows what they’re doing and can deliver it in the right way, then it’s beneficial. If it’s somebody that goes to Google to get some stuff, you know, that you can see can try by the kids on a Sunday morning…”* (FG3, P11)

This reiterates the importance of the coach, their qualifications and experience, and their actions in shaping parental perceptions of how strength and conditioning might be best implemented in youth sport. Parents also acknowledged that it would be relevant for the children to understand why they are doing strength and conditioning training and the related benefits. As examples:

*“*[coaches] *need to make it quite rewarding for them so that they feel they’ve got something out of it. So I think as, as the sessions develop, they’ll get more balanced...”* (FG2, P6)

And:

*“anything improves in football, I think that’s probably that’s what they’d want to see, isn’t it? To recognise that you’re much better at that because you’re doing this”* (FG2, P8)

Parents also identified two other areas that should be considered when implementing strength and conditioning with children, namely resilience and autonomy. In regard to resilience, parents noted that children tend to give up more easily when facing challenging activities, which may be a constraint when implementing strength and conditioning in their training activities. For example, one parent noted:

*“when they first start feeling this is a bit difficult. So the pushing through it, just stop”* (FG1, P1)

One potential facilitator to implementing strength and conditioning with children which parents suggested was in developing children’s autonomy. Comments relating to autonomy were highlighted in all of the focus groups, for example:

*“They are kids but it’s giving them ownership of what they’re doing. We’re not standing there saying you’re doing this. That’s taking ownership of it. Whether they want to do it or not, is their choice”* (FG3, P13)

And:

*“What do they want? Yeah, well and giving them a bit of the ownership of it about they feel that they need that”* (FG4, P21)

## 4. Discussion

The current study provides a unique insight into parental perceptions on use of strength and conditioning in children and youth. While there are empirical research studies that have examined the benefits of undertaking strength and conditioning in children [4,5,6], their remain considerable barriers to children and young people undertaking strength and conditioning with the majority of these based on myths and misconceptions relating to children’s participation in strength training [14]. The current study addresses a key gap in the literature, namely understanding the beliefs and perceptions of parents in relation to their children undertaking strength and conditioning. As parents are critical influences in children’s lives, particularly in relation to exercise and sport [18,19], understanding how they feel about strength and conditioning is an essential step in understanding how strength and conditioning can be implemented in an acceptable manner to benefit children’s health and sport and exercise participation.

The focus group interviews revealed a number of themes, some of which highlighted some of the established misconceptions regarding strength and conditioning in children and youth. For example, there were some parents who cited aspects related to growth as a consideration for their child undertaking strength and conditioning. This aligns with Faigenbaum et al. [14] assertion that parent may hold negative views on strength and conditioning due to misconceptions that it retards growth. However, in the present study, the parents’ views were more nuanced, citing concerns around growth but acknowledging that it depends on education and training of the coach administering the strength and conditioning practice. It was perhaps surprising that the commonly cited misconceptions of engaging in strength and conditioning in children were not cited more often given the suggestions of Faigenbaum et al. [14] regarding parental reticence to engage in such activity due to these myths. Likewise, fathers appeared to have greater knowledge and experience around the topic of strength and conditioning compared to mothers.

The coach delivering any strength and conditioning was a key factor in two themes from the focus group interviews. This is not surprising given that the coach is central to delivering youth sport for the parents involved in this study, and that prior research documenting the views of the children themselves has also suggested the individual delivering strength and conditioning was a key influence for adolescent boys in their engagement in strength training [15]. While the work by Cox et al. [15] is useful in highlighting boys’ perceptions of strength and conditioning type exercise, it did not delve into issues around differential developmental needs or the individuals delivering any strength and conditioning intervention. The present study elucidates this issue and this should be considered as key consideration arising from the findings of the present study. Parents were keen that any coach delivering strength and conditioning have appropriate education/qualifications to deliver such, in addition to standard sports coaching and that communication with the coach was essential to help parents understand what strength and conditioning activities are being conducted with their child and what the benefits of such are. Once interesting observation from the current study and relating to effective implementation of strength and conditioning was a difference in how parents saw strength and conditioning being implemented that was dependent on age band. Parents of children playing grassroots soccer at under 12 age and below suggested that, to be effective, strength and conditioning might have to be ‘hidden’ within their children’s soccer training sessions, whereas parents of children playing grassroots soccer at under 13 age and above suggested that, to be effective, strength and conditioning might need to be considered as a separate session/activity outside of their child’s soccer training sessions. Notably, parents of children at under 13s age and above did observe that their children were already engaged with some forms of strength and conditioning, unsupervised and likely incorrectly as gym memberships are available from the age of 14 upwards in the UK. This is an interesting observation as it maps somewhat to the change in structure of grassroots soccer for youth in England [31], where at Under 12 age band and younger, players undertake mini-soccer, comprising smaller sided games up to 9 vs. 9 player format. Conversely, for Under 13 age band and above players move to the standard 11 vs. 11 format which also comprises the adult game. Notably, parents identified that children in the older age band (Under 13s and older) were already engaged in some forms of Strength and Conditioning but were perhaps not fully aware or competence in how to perform resistance exercise appropriately. Similarly, the split by age band identified by parents in the current study also marks the move from childhood to adolescence and might suggest that as children move into older adulthood/adolescence that an approach to strength and conditioning implementation which is appears more formal and gym based might be more successful, while for younger ages implementing strength and conditioning into their sport activity in a more integrated manner would result in better success, at least from the parental perspective. Parental suggestions regarding children having ownership of strength and conditioning activity also align well with the tenants of self-determination theory [32] and offer a coach friendly mechanism to help children engage and adhere to strength and conditioning in particular.

Collectively, the view of parents represented in the current study provide some useful guidance for professionals working in public health, education, or coaches in community sport to maximise the possibility of any strength and conditioning intervention being successful in terms of recruitment and implementation. In particular:

- Provide education to parents on what strength and conditioning is and how it might benefit their child. This may need to be targeted to those parents who have less awareness of strength and conditioning as a concept.
- Provide education on the benefits of strength and conditioning for children specifically, with an emphasis on who it links back to their chosen sport(s)
- Implementation of strength and conditioning needs to be differentiated with older children having more formalised sessions and with younger children, sessions which are integrated into their sports activities.
- Create a dialogue between parents and coaches to inform the parents what strength and conditioning activities are being undertake in coaching sessions and why, alongside individual feedback regarding their child. For example, this might be a newsletter that could be sent home, email, social media update, or even a conversation.

There are of course limitations to the present study. Our sample included parents of children who were regularly engaged in team sport at grassroots levels. As such, the sample represents parents of children who might be considered ‘sporty’. An assumption could be made that such parents would broadly hold positive views regarding sport and exercise for their child due to their involvement in grassroots sport. We are also cognisant that our sample represents parents of boys and this might be considered a limitation. Certainly, future work examining perceptions of strength and conditioning in both parents of girls and girls themselves would be useful, the focus on boys in the present study was deliberate. Prior work [15] has suggested that boys might respond more positively to interventions that target improved muscular strength, which is the prime use of strength and conditioning. Parental perceptions of strength and conditioning for boys specifically has not been undertaken, despite work examining the perceptions of boys themselves [15] and scientific evidence examining the effects of resistance exercise in this populations [1.2.7]. Consequently, our focus on boys closes the loop for researchers and practitioners. Boys themselves may view undertaking strength and conditioning in a positive manner [15] and the evidence might suggest strength and conditioning is safe and beneficial for boys to undertake [14], but if parents do not view strength and conditioning positively, or hold misconceptions about its use, the implementation of strength and conditioning practice for boys is unlikely to be successful [14,15]. It is useful to recognise that the views of the children themselves were not gained in the current study, and the parents involved in the focus groups suggested it would be good to also hear the views of the children themselves when considering how to best implement strength and conditioning intervention for this group. This should be a key future research focus. A further consideration is that only audio recordings were obtained and thus we were unable to examine the non-verbal language displayed during the focus groups in gaining further depth to the data obtained.

## 5. Conclusions

The results of the present study suggest that parents of children who play grassroots sport hold generally positive perceptions of strength and conditioning in general and the use of strength and conditioning in children as beneficial for both physical and mental development. Key factors relating to successful implementation of strength and conditioning in children’s grassroots sport focus on having a qualified coach in that particular area (rather than a generic sports coach), effective communication between the coach and parents, and coach and children in terms of why they would engage with strength and conditioning, in addition to the benefits of strength and conditioning for the child’s overall sports performance. Parents also identified tailoring the nature and approach of strength and conditioning activity relative to their child’s developmental need, and also considering their physical competence as being important for successful implementation. This assertion is based on the noted differences in approaches parents suggested would be effective for children at under 13s level or younger compared to those older than under 13s level. More formalised, gym-based approaches, undertaken in addition to the child’s sports training being considered more attractive for older children (>12 years age) and more integrated game-based approaches being considered attractive for younger children (<12 years age).

## Figures and Tables

**Table 1:** Interview guide.

Interview Guide
*Ice breaker activity* Each focus group was shown a short (time) video of an integrated neuromuscular training session for children. This was taken from the published work of Fort-Vanmeerhaeghe et al. [24] which included in its publication video resource outlining approaches to strength and conditioning in children and youth and was used as a base to provide a shared baseline understanding of what Strength and conditioning for youth sport participants comprises. **Topic 1: Parents knowledge and beliefs about strength and conditioning** 1. When you hear the word strength or conditioning—what does this mean to you? if parents had no knowledge of the word—video prompt used. 2. How do you feel about strength and conditioning training in children? Prompts: perceived benefits/concerns **Topic 2: Parents perceptions about the implementation of strength and conditioning with their child** 1. How would you feel about the implementation of strength and conditioning within your child’s training practice? 2. If the coach was to implement strength and conditioning with your child what would you need? Prompts: practicalities, challenges

**Figure A1:**
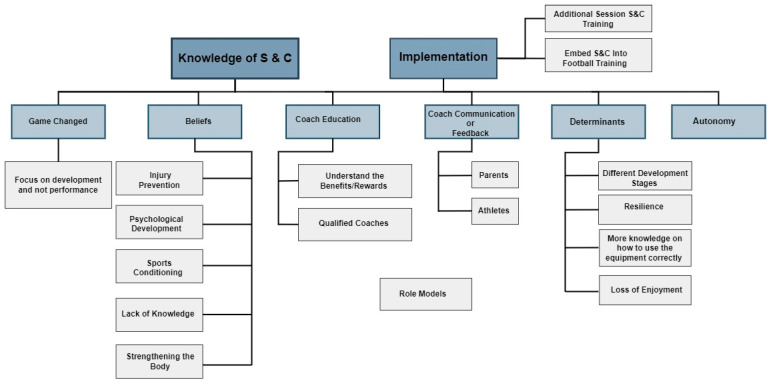
Coding tree for themes and subthemes arising from the focus group interviews.

## Data Availability

Data regarding this cubmission can be provided upon reasonable request to the primary author.

## References

[1] Stricker P., Faigenbaum A. (2020). McCambridge, T. Council on Sports Medicine and Fitness. Resistance training for children and adolescents. Pediatrics.

[2] Smith J.J., Eather N., Morgan P.J., Plotnikoff R.C., Faigenbaum A.D., Lubans D.R. (2014). The Health Benefits of Muscular Fitness for Children and Adolescents: A Systematic Review and Meta-Analysis. Sports Med..

[3] Duncan M.J., Eyre E., Oxford S.W. (2018). The Effects of 10-week Integrated Neuromuscular Training on Fundamental Movement Skills and Physical Self-efficacy in 6-7-Year-Old Children. J. Strength Cond Res..

[4] Torres-Costoso A., López-Muñoz P., Martínez-Vizcaíno V., Álvarez-Bueno C., Cavero-Redondo I. (2020). Association Between Muscular Strength and Bone Health from Children to Young Adults: A Systematic Review and Meta-analysis. Sports Med..

[5] Bea J.W., Blew R.M., Howe C., Hetherington-Rauth M., Going S.B. (2017). Resistance Training Effects on Metabolic Function Among Youth: A Systematic Review. Pediatr. Exerc. Sci..

[6] Moliner-Urdiales D., Ruiz J.R., Vicente-Rodriguez G., Ortega F.B., Rey-Lopez J.P., Espana-Romero V., Casajus J.A., Molnar D., Widhalm K., Dallongeville J. (2009). Associations of muscular and cardiorespiratory fitness with total and central body fat in adolescents: The HELENA Study. Br. J. Sports Med..

[7] Collins H., Booth J.N., Duncan A., Fawkner S., Niven A. (2019). The Effect of Resistance Training Interventions on ‘The Self’ in Youth: A Systematic Review and Meta-analysis. Sports Med. Open.

[8] Faigenbaum A.D., Kraemer W.J., Blimkie C.J., Jeffreys I., Micheli L.J., Nitka M., Rowland T.W. (2009). Youth resistance training: Updated position statement paper from the National Strength and Conditioning Association. J. Strength Cond. Res..

[9] Stodden D., Langendorfer S., Roberton M.A. (2009). The association between motor skill competence and physical fitness in young adults. Res. Q. Exerc. Sport.

[10] Bull F.C., Al-Ansari S.S., Biddle S., Borodulin K., Buman M.P., Cardon G., Carty C., Chaput J.P., Chastin S., Chou R. (2020). World Health organization 2020 guidelines on physical activity and sedentary behaviour. Br. J. Sports Med..

[11] (2019). Physical Activity Guidelines: UK Chief Medical Officers’ Report—GOV.UK. https://www.gov.uk/government/publications/physical-activity-guidelines-uk-chief-medical-officers-report.

[12] Bennie J.A., Faulkner G., Smith J.J. (2022). The epidemiology of muscle-strengthening activity among adolescents from 28 European countries. Scand. J. Public Health.

[13] Dooley F.L., Kaster T., Fitzgerald J.S., Walch T.J., Annandale M., Ferrar K., Lang J.J., Smith J.J., Tomkinson G.R. (2020). A Systematic Analysis of Temporal Trends in the Handgrip Strength of 2,216,320 Children and Adolescents Between 1967 and 2017. Sports Med..

[14] Faigenbaum A.D., Stracciolini A., MacDonald J.P., Rial Rebullido T. (2022). Mythology of youth resistance training. Br. J. Sports Med..

[15] Cox A., Fairclough S.J., Noonan R.J. (2021). “It’s Just Not Something We Do at School”. Adolescent Boys’ Understanding, Perceptions, and Experiences of Muscular Fitness Activity. Int. J. Environ. Res. Public Health.

[16] Knight C.J., Berrow S.R., Harwood C.G. (2017). Parenting in sport. Curr. Opin. Psychol..

[17] Frosch C.A., Schoppe-Sullivan S.J., O’Banion D.D. (2021). Parenting and Child Development: A Relational Health Perspective. Am. J. Lifestyle Med..

[18] Bonavolontà V., Cataldi S., Latino F., Carvutto R., De Candia M., Mastrorilli G., Messina G., Patti A., Fischetti F. (2021). The Role of Parental Involvement in Youth Sport Experience: Perceived and Desired Behavior by Male Soccer Players. Int. J. Environ. Res. Public Health.

[19] Edwardson C.L., Gorely T. (2010). Activity-related parenting practices and children’s objectively measured physical activity. Pediatr. Exerc. Sci..

[20] Tong A., Sainsbury P., Craig J. (2007). Consolidated criteria for reporting qualitative research (COREQ): A 32-item checklist for interviews and focus groups. Int. J. Qual. Health Care.

[21] FIFA (2011). Grassroots Football. Zurich: FIFA. https://www.fifa.com/who-we-are/news/grassroots-goes-live-fifa-com-1462069.

[22] McNamara C. (2009). General Guidelines for Conducting Interviews. http://managementhelp.org/evaluatn/intrview.htm.

[23] Turner D.W. (2010). Qualitative Interview Design: A Practical Guide for Novice Investigators. Qual. Rep..

[24] Fort-Vanmeerhaeghe A., Romero-Rodriguez D., Lloyd R.S., Kushner A., Myer G.D. (2016). Integrative neuromuscular training in youth athletes. Part II: Strategies to prevent injuries and improve performance. Strength Cond. J..

[25] Creswell J.W. (2007). Qualitative Inquiry and Research Design: Choosing among Five Approaches.

[26] Braun V., Clarke V. (2006). Using Thematic Analysis in Psychology. Qual. Res. Psychol..

[27] Braun V., Clarke V., Weate P., Smith B., Sparkes A.C. (2016). Using thematic analysis in sport and exercise research. Routledge Handbook of Qualitative Research in Sport and Exercise.

[28] Lincoln Y.S., Guba E.G. (1985). Naturalistic Inquiry.

[29] Smith B., McGannon K.R. (2018). Developing rigour in qualitative research: Problems and opportunities within sport and exercise psychology. Int. Rev. Sport Exerc. Psychol..

[30] Guest G., Bunce A., Johnson L. (2006). How many interviews are enough? An experiment with data saturation and variability. Field Methods.

[31] The FA (2022). The FA Handbook 2022-23. Legal and Governance Division.

[32] Deci E.L., Ryan R.M. (2000). The “what” and “why” of goal pursuits: Human needs and the self-determination of behavior. Psychol. Inq..

